# Incidence, Associated Factors, and Behavioral Consequences of Intraoperative Awareness and Dreams During General Anesthesia Among Adult Patients: A Prospective Cohort Study

**DOI:** 10.7759/cureus.62843

**Published:** 2024-06-21

**Authors:** Roudha Al Jabri, Jyoti Burad, Al Muayad Al Moosa

**Affiliations:** 1 Anesthesia and Intensive Care, Sultan Qaboos University Hospital, Muscat, OMN

**Keywords:** nightmares, neuromuscular blocking agents, behavioral symptoms, general anesthesia, dreams, intraoperative awareness

## Abstract

Background

The intraoperative awareness with recall phenomenon involves the memory of intraoperative events. The reported incidence is 0.001%. Awareness is rare intraoperatively but has profound implications. This research aimed to study intraoperative awareness and dream experiences in the Omani population undergoing general anesthesia.

Methods

This prospective cohort study involving 300 adult patients was conducted at a tertiary-level hospital between July and December 2021. Awareness was assessed in the post-anesthesia care unit and then at home on the seventh day and one month telephonically using a modified Brice questionnaire. The study's primary outcome was the incidence of IOA and dreams in adult Omani patients undergoing GA. The secondary outcome was identifying the factors associated with intraoperative awareness and dreams under GA.

Results

In this study, the incidence of awareness was 0.3% while 7.6% of patients reported intraoperative dreams. The patient who experienced IOA underwent an endoscopic retrograde cholangiopancreatography under GA with neuromuscular blockade and had a prolonged recovery. A total of 49.33% developed psychological effects postoperatively; anxiety, irritability, fear of surgery, change in memory, and repetitive nightmares were the most common ones. Because of the limited sample size, no factor associated significantly with these outcomes was found.

Conclusion

This study observed a higher incidence of intraoperative awareness and dreams. Awareness might be due to prolonged recovery and the use of neuromuscular blocking agents, but a focused study is required to confirm this finding. The incidence of intraoperative dreams and postoperative psychological effects of GA was also high. It may be worth exploring these findings with larger population-based research.

## Introduction

Intraoperative awareness (IOA) is rarely reported after general anesthesia (GA), and the extent of the recollections varies from vague to explicit [1]. Several situations can make the doctor inadvertently deliver light anesthesia. Certain operations require light anesthesia, like cesarean section, which increases awareness by about 0.4% [2]. In other cases, the patient may be unable to tolerate a sufficient anesthetic dose due to many reasons such as poor cardiac function, severe hypervolemia, unstable hemodynamics, hypothermia, or acute intoxication. IOA can be higher in females, specific procedures, repeated intubation attempts, hypovolemia, hyperthermia, total intravenous anesthesia (TIVA), inadequate anesthesia, and neuromuscular blockers [2,3].

IOA can lead to complications like persistent nightmares, behavioral changes, and personality changes. Intraoperative awareness can lead to posttraumatic stress disorder (PTSD), affecting the everyday life of patients. In this era of early recovery after anesthesia, a delay in recovery and return to normal life is an important issue to address. Hence, it is crucial to study IOA. This study investigated the incidence, risk factors, and consequences of IOA and dreams in Omani patients undergoing GA.

## Materials and methods

This prospective, closed cohort study was conducted at a tertiary-care teaching hospital. The Institutional Medical Research Ethics Committee of Sultan Qaboos University Hospital granted ethical approval for this study. All adult patients (more than 14 years of age) scheduled for elective and emergency surgery under GA with an ASA (American Society of Anesthesiologists) score of 1-3 were approached, and those who consented were included in the study. Those who refused consent were not oriented preoperatively and had neurological conditions like dementia, Alzheimer’s disease, psychosis, depression, stroke, head injury, or other known cognitive impairments were auditorily and vocally impaired, and those who received only regional anesthesia were excluded.

After inclusion, the patient's demographic data was noted. The patients received general anesthesia as per standard institutional protocols. Once the surgery finished, the patients were shifted to the PACU. Awareness was assessed when the patient was fully awake before discharge from PACU through a face-to-face interview. The patients were followed further at two points post-surgery (on the seventh and thirtieth days) by telephone. A modified Brice questionnaire was used to question the patients, as it has been validated for an assessment of awareness during GA [4]. Intraoperative details like type of surgery and GA (inhalational or total intravenous anesthesia (TIVA)), duration of surgery, minimum alveolar concentration (MAC) value, GA medications, vitals, shock development, and questionnaire responses were recorded.

The study's primary outcome was the incidence of intraoperative awareness and dreams during GA in the adult Omani population. The secondary outcome was the determination of etiological factors of intraoperative awareness under GA. All patients included in the study were exposed to GA (either inhalational or TIVA). Potential confounders were age, gender, comorbidities, drug history, type of surgery, and ASA severity grading. All the data were obtained from the anesthesia records in the attached electronic patient record (EPR) documents. The patient's identity was veiled with codes, and data were statistically analyzed.

Bias could have been introduced, as it is an observational study, and the anesthesia was not the same for all. However, the department uses protocols when administering anesthesia. Second, awareness was assessed using a well-validated questionnaire.

Sample size

As this was time-based research, all adult patients posted for GA were approached for inclusion in the study. Those who consented were included during the five-month study period, and the patients were followed up for one month each.

Statistical methods

SPSS software (version 25; IBM Corp., Armonk, NY, US) was used to code and analyze individual data. The data for intraoperative awareness were presented using descriptive statistics. For the outcome of dreams, a chi-square test was performed to determine the relationship between the various categorical variables and dreams, with a p-value of <0.05 being statistically significant.

## Results

**Figure 1:**
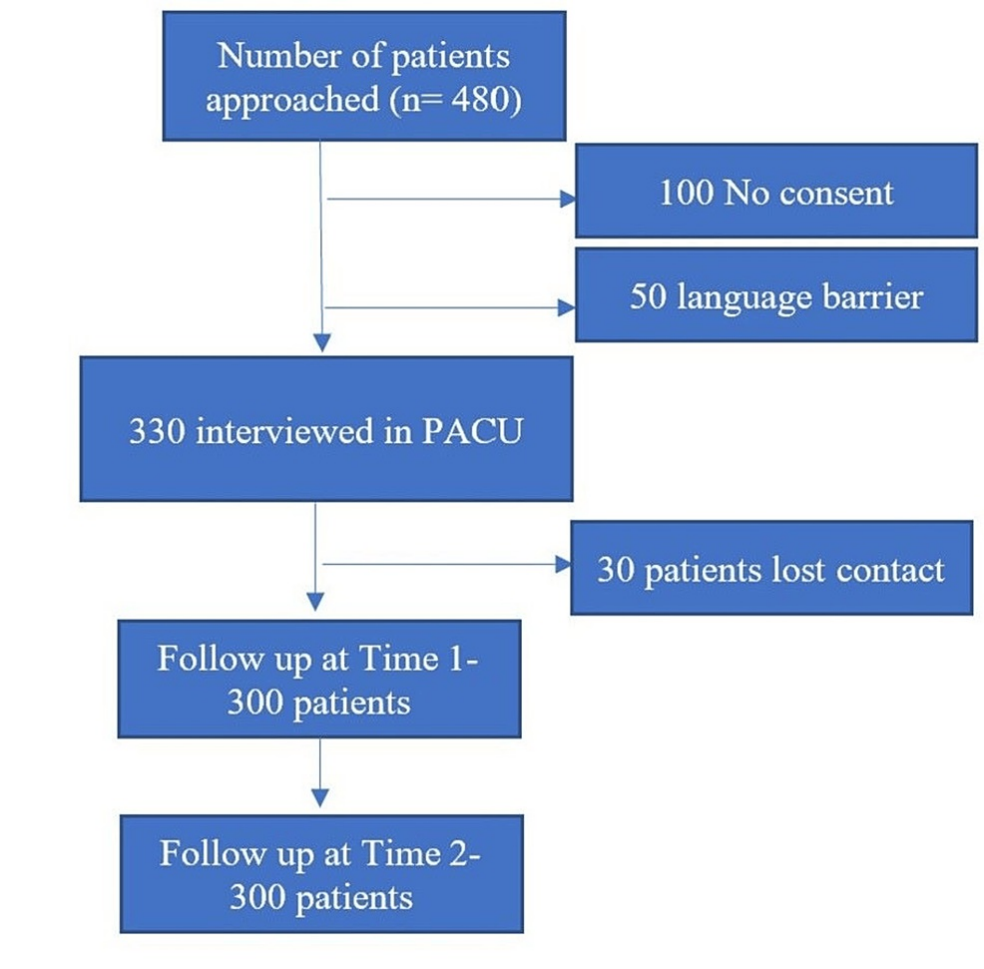
Flow of cases during the study PACU: post-anesthesia care unit; Time 1: postoperative day 7; Time 2: postoperative day 30

We attempted to recruit all the adult Omani patients (450) who underwent surgery under GA within the study period. There was no consent for 100 patients, and they were excluded. There were 330 valid responses to the questionnaires at the time of discharge from the PACU. After the initial enrollment, efforts were made to follow up with all participants at home twice. Participants (30/330, 9%) who became unreachable despite repeated attempts for contact were excluded from the analysis. Three hundred valid surveys were studied and included in the final analysis, as shown in Figure 1.

Most participants were female (210/300, 70%). The overall mean age of patients was 39.54 years. About two-thirds (189/300, 63%) had general surgery. At the same time, only 0.34% (1/300) underwent neurosurgery. Moreover, for most types of surgeries (274/300, 91%), patients received inhalation anesthesia, and in more than half of patients, their MAC value was more than one while 9% (26/300) received total intravenous anesthesia (TIVA) (Table 1).

**Table 1:** Patient characteristics SD: standard deviation; ENT: ear, nose, throat; TIVA: total intravenous anesthesia; MAC: minimum alveolar concentration; ASA: American Society of Anesthesiologists The data have been presented as mean (SD) for age and n (%) for the rest of the variables.

Variables		n (%)
Age (years) Mean ± SD	39.54 ± 15.93	NA
Gender	Male	90 (30)
	Female	210 (70)
Type of surgery	General	189 (63)
	Gynecologic	59 (20)
	Urology	8 (3)
	ENT	21 (7)
	Orthopedic	22 (7)
	Neurological	0.34 (1)
Type of anesthesia	Inhalation	274 (91)
	TIVA	26 (9)
MAC value	<1	104 (35)
	>1	169 (56)
ASA grade	1	76 (25)
	2	185 (62)
	3	39 (13)
Premedication (benzodiazepine)	Yes	109 (36)
Presence of shock during surgery	Yes	12 (4)
Patient comorbidities	None	220 (73)
	HTN	36 (12)
	DM	23 (8)
	IHD	3 (1)
Long-term use of opioids	Yes	3 (1)

The incidence of unintended awareness during GA in our research was 0.3%. The awareness occurred in a female patient in her 20s, ASA status I operated for elective endoscopic retrograde cholangiopancreatography (ERCP). She had a past surgical history of a gastric sleeve operation nine months prior and had a body mass index of 21.3. The patient did not receive benzodiazepine preoperatively. General anesthesia was induced with 2.2 mg/kg of propofol, and a neuromuscular blocker was initiated with 0.1 mg/kg of cisatracurium. She was intubated and proned for the procedure. Maintenance of anesthesia was achieved with oxygen, air, and sevoflurane. During the case, there was no hypotension. The MAC value was maintained at more than one throughout, and bi-spectral index (BIS) monitoring was not performed. The duration of the surgery was two hours. At the end of the surgery, the patient was turned supine, and inhalational anesthetics were stopped. She was given a reversal for the neuromuscular blockade. However, her recovery was delayed by 20 minutes (Table 2).

**Table 2:** Primary outcome: incidence of intraoperative awareness and dreams Data presented as n (%).

Outcomes	n= 300, n (%)
Awareness with recall	1 (0.333)
Patients with intraoperative dreams	23 (7.7)

Dreams were more commonly reported after surgery than awareness. Overall, 7.7% (23/300) of patients reported dreaming during the interviews. Furthermore, the males had a higher incidence of intraoperative dreams as compared to females (8.9%, 8/90 versus 7.1%, 15/210). And 7.9% (15/191) of patients who did not receive premedication (benzodiazepine) experienced dreams compared to 7.3% (8/109) of those who received it (P=0.872). In most cases, dreams experienced by the patient were pleasant and had some meaning to them (e.g., they involved family, friends, work, and recreation). There were very few unpleasant dreams. Frequently, patients reported dreaming just before they awoke, and some even reported that their anesthesiologist "tried to wake them up" while they were dreaming. No significant difference was found between intraoperative awareness and gender, type of anesthesia, MAC value, and premedication administration (Table 3).

**Table 3:** Secondary outcome: factors associated with intraoperative dreams P<0.05 was considered as significant. SD: standard deviation; TIVA: total intravenous anesthesia; MAC: minimum alveolar concentration; BDZ: benzodiazepine; ENT: ear, nose, throat

Variables		Dream n (%)	P-value	Odds ratio	95% CI
Age (years) Mean ± SD		38.48+12.3	NA	NA	NA
Gender	Male	8 (8.9)	0.602	0.788	0.32-1.93
	Female	15 (7.1)			
Type of anesthesia	Inhalation	21 (7.7)	0.996	1.004	0.22-4.54
	TIVA	2 (7.7)			
MAC	>1	14 (8.3)	0.641	0.111	-0.27-0.17
	<1	7 (6.7)			
Premedication (BDZ)	Yes	8 (7.3)	1.00	0.929	0.38-2.27
Shock	Yes	1 (8.3)	0.609	1.147	0.14-9.3
Long-term analgesics/ BDZ medications	Yes	0 (0)	NA	NA	NA
Type of surgery	General (n=189)	18 (9.5)	NA	NA	NA
	Gynecological (n=59)	2 (3.4)			
	Orthopedic (n=22)	0 (0)	NA	NA	NA
	ENT (n=21)	2 (9.5)	NA	NA	NA
	Urology (n=8)	1 (12.5)	NA	NA	NA
	Neurological (n=1)	0 (0)	NA	NA	NA

A total of 148 out of 300 (49.33%) patients developed behavioral changes post-surgery, including anxiety, irritability, fear of surgery (tomophobia), change in memory, and repetitive nightmares. The overall mean age of patients who developed behavioral changes postoperatively was 39.11 years old. Our result demonstrated that females (118/60.2%) are more likely to develop behavioral changes postoperatively (p-value: 0.00, OR: 2.6, CI 95% 1.57-4.54) (Table 4).

**Table 4:** Factors associated with behavioral changes The patients with behavioral changes are represented as n (%). A p-value of < 0.05 is considered as significant. SD: standard deviation; TIVA: total intravenous anesthesia; MAC: minimum alveolar concentration; BDZ: benzodiazepine; ENT: ear, nose, throat

Variables		Behavior change n (%)	P-value	Odds ratio	95% CI
Age (years) mean ± SD		39.11+14.6	NA	NA	NA
Gender	Male	30 (36.1)	0.00	2.673	1.57-4.54
	Female	118 (60.2)			
Type of anesthesia	Inhalation	138 (54.1)	0.287	0.606	0.25-1.41
	TIVA	10 (41.7)			
MAC	>1	75 (48.1)	0.018	0.061	0.25-0.265
	<1	62 (63.3)			
Long-term analgesics/BDZ medications	Yes	3 (100)	0.250	0.525	0.47-0.58
Premedication (BDZ)	Yes	45 (46.4)	0.131	0.664	0.41-1.08
Intraoperative shock	Yes	3 (27.3)	0.123	0.323	0.08-1.24
Type of surgery	General (n=170)	73 (42.9)	NA	NA	NA
	Gynecological (n=59)	44 (74.6)			
	Orthopedic (n=20)	15 (75)			
	ENT (n=21)	11 (52.4)			
	Urology (n=8)	4 (50)			
	Neurological (n=1)	1 (100)			
Intraoperative awareness	Yes	1 (100)	1.000	0.529	0.47-0.59
Intraoperative dreams	Yes	14 (60.9)	0.516	1.416	0.59-3.38

## Discussion

In this study, the incidence of intraoperative awareness was 0.3%, and that of dreams was 7.6%. Our case had a delayed recovery after the surgical procedure. No inhalational anesthetic was given during recovery, and the muscle relaxant effect persisted. This could have caused inadequate depth of anesthesia with paralysis, causing awareness. This is a very common scenario that can happen to any patient globally.

However, our awareness rate is higher than in a previously reported study [5]. It has been shown that certain patient-related risk factors like gender may be associated with IOA, as females are believed to recover faster from anesthesia. Our study, however, does not establish a significant relationship between IOA and gender due to the limited number of cases. Nonetheless, other studies have reported conflicting results, with higher incidences of IOA, sometimes in males and at other times in females [6,7]. A systematic review shows that females exhibited a higher incidence of awareness with postoperative recall (33 studies, OR 1.37, 95% CI 1.09 to 1.75) and consciousness during anesthesia (3 studies, OR 2.09, 95% CI 1.04 to 4.23) as compared to males. Moreover, females demonstrated faster time to emergence, including time to eye-opening (10 studies, mean differences -2.28 min, 95% confidence intervals -3.58 to -0.98), and time to response to command (6 studies, mean differences -2.84 min, 95% confidence intervals -4.07 to -1.62) as compared to males [8]. A particular group of patients can be more resistant to anesthetic drugs than others. The reason why some patients require a higher dose still needs to be apparent. Concurrent medications can also adversely influence anesthetic agents' distribution and metabolism [9]. The 5th National Audit Project (NAP5) study reported that 1.6% of patients with accidental awareness during anesthesia had a previous history of IOA [10].

Neuromuscular blockade is a risk factor for awareness during surgery. In a study that included 11,785 patients undergoing GA, awareness was noted in 0.1% of cases for whom no muscle paralysis was administered and 0.18% receiving the neuro-muscular blockade [6]. Regarding our positive case series, neuromuscular blocking agents can mask patient motion, which would probably provide an early clinical sign of light anesthesia. In the United Kingdom, findings from NAP5 showed 1 in 8000 incidences of accidental awareness after general anesthesia in cases where an NMB agent was used, compared with approximately 1 in 136,000 when no NMB agent was used. The use of neuromuscular agents has increased the risk of awareness during GA [10].

Another risk factor of awareness is light anesthesia, which happens when a patient is awakened at the end of the surgery, just like our patient. It has also been reported with GA for cesarean section (0.4%), where light anesthesia is required at the start of surgery till the delivery of the baby to minimize respiratory depression in newborns. Other situations where light GA is frequently observed are cardiac surgery and surgeries with a high risk for hemodynamic decompensation. However, it is interesting to note that no cases of awareness were found in obstetric patients in this study, agreeing with previous studies that observed no higher rates of awareness in obstetric patients [11].

Our case with IOA did not receive the benzodiazepine premedication, which may be a risk factor for awareness. This observation disagrees with the study of 1500 outpatients and 2343 inpatients, showing that the use of benzodiazepine premedication predominantly was significantly more prevalent among inpatients compared to outpatients (88% versus 15%; P < 0.001). However, when analyzing data from both inpatients and outpatients together, there was no observed variance in the occurrence of awareness and recall between patients who received benzodiazepine premedication and those who did not [12]. However, some researchers recommended using benzodiazepine when low doses of anesthetics are administered [3-13].

Dreams during anesthesia are common but remain poorly understood. Dreams may be related to low doses of anesthesia and represent near awareness. In this study, the incidence of dreams among patients was 7.6%. This observation agrees with a study of 300 patients and illustrates that almost all dreams resembled dreams of rapid eye movement sleep (usual dreams), except that they were strange and shorter [14]. A study of 2251 patients found that females more often report dreams post-operative than males. A possible explanation is that females emerge from anesthesia faster than males and can communicate their dreams earlier before they are forgotten [15]. Another study showed that 36% of men had reported dreaming, whereas, only 20% of women reported dreaming after surgery [16]. Our study shows no significant association between intraoperative dreams and sex (p>0.219). Moreover, a prior study found that propofol maintenance was associated with higher incidences of dreaming than enflurane, isoflurane, sevoflurane, or desflurane. A possible explanation for that is that patients receiving propofol maintenance emerged more rapidly from anesthesia than patients receiving enflurane or isoflurane [17].

Some patients who have experienced IOA may progress without psychological disorders. One of the most feared complications is PTSD. This kind of psychological disorder may be experienced in a stressful situation with a functional, psychic, and social impairment that may persist for a long time and, if not adequately treated, develop into a chronic disorder in 25% of the cases. A person experiencing PTSD exhibits difficulty sleeping steadily, anxiety, concentration and humor disorders, irritability, fear of anesthesia, depression, and nightmares. In contrast, a prospective cohort study conducted in Canada and the United States shows that only 20.1% of patients had PTSD [18]. PTSD can affect a person's ability to work, carry out daily activities, or relate to friends and family. Psychotherapy, medications, or both are the primary treatment.

Many preventive strategies have been recommended for Intraoperative awareness. Premedication with an amnesic (benzodiazepine) is recommended as a preventive measure, especially when light anesthesia is expected. The efficacy of prophylactic administration of midazolam as an adjuvant during TIVA has been evaluated in one double-masked, randomized clinical trial; this study reported a lower incidence of IOA as compared to the placebo group. The administration of benzodiazepine should be made on patients who require light doses of anesthetics and those undergoing trauma surgery, emergency surgery, cardiac surgery, or TIVA. On the other hand, delayed emergence can accompany the use of benzodiazepines [19,20].

Thorough checking of the anesthesia delivery system before induction of anesthesia should be mandated, as cases have been linked to low anesthetic concentrations leading to IOA. In a study, 8372 cases were reported to the Anesthetic Incident Monitoring System, identifying 81 cases for which the perioperative recall matched awareness. In 16 of these patients, failure to deliver volatile anesthetic resulted in awareness, while in 32 cases, a medication error resulted in unintentional paralysis of a conscious patient. Regularly checking the anesthesia machine, including the circuit and vaporizer, monitoring the inhalational drugs and concentrations of inspired and expired gases, and administering an anesthetic infusion via a dedicated intravenous line are all basic precautions that can help prevent awareness [3-20]. BIS monitoring demonstrating the brain’s electrical activity can be used to monitor the anesthesia depth, and this purpose is advocated mainly for TIVA. This was not routinely used for our patients.

Strength of the study

We used the modified Brice questionnaire, the gold standard for prospective observational studies for eliciting the IOA.

Limitations

This study had many limitations. First, there was difficulty in getting patient responses in the PACU because of the sedative effect. Second, the number of elective surgeries declined because of the COVID-19- pandemic was low; therefore, a future study with a large sample size is needed further to evaluate the relationship between IOA and their risk factors. Furthermore, the study did not monitor the depth of anesthesia due to the unavailability of a BIS. In addition, age-adjusted MAC values were not monitored, which may have impacted the accuracy of our results. We also acknowledge that not all risk factors were considered, potentially introducing confounding bias. Finally, this research's scope included only one tertiary center so that the results may be representative of only some of the population locally or across the world.

## Conclusions

In this research, the incidence of intraoperative awareness during GA was high (0.33%). Dreams were frequently reported after surgery (7.6%). None of the factors, like the type of general anesthesia, MAC, gender, and premedication, significantly affected the incidence of dreams. Studies with a larger sample size are needed to highlight their effect. A dedicated study with longer follow-up is needed to elicit PTSD and its effect on patient's return to daily life.

## Appendices

**Table 5:** Modified Brice Questionnaire

What is the last thing you remember before going to sleep (please tick one box)?	-Being in the pre-op area ☐ -Seeing the operating room ☐ -Being with family ☐ -Hearing voices ☐ -Feeling mask on face ☐ -Smell of gas ☐ -Burning or stinging in the IV line ☐ -Other [Please write below]:
What is the first thing you remember after waking up (please tick one box)?	-Hearing voices ☐ -Feeling breathing tube ☐ -Feeling mask on face ☐ -Feeling pain ☐ -Seeing the operating room ☐ -Being in the recovery room ☐ -Being with family ☐ -Being in ICU ☐ -Nothing ☐ -Other [Please write below]:
Do you remember anything between going to sleep and waking up (please tick box)?	-No ☐ -Yes: -Hearing voices ☐ -Hearing events of the surgery ☐ -Unable to move or breathe ☐ -Anxiety/stress ☐ -Feeling pain ☐ -Sensation of breathing tube ☐ -Feeling surgery without pain ☐ -Other [Please write below]:
Did you dream during your procedure (please tick box)?	-No ☐ -Yes ☐
What was the worst thing about your operation (please tick box)?	-Anxiety ☐ -Pain ☐ -Recovery process ☐ -Unable to carry out usual activities ☐ -Awareness ☐ -Other [Please write below]:
Do you have a bad dreams\nightmares?	-No ☐ -Yes ☐
Were there any behavioral changes 1 week after surgery?	-Irritability ☐ -repetitive nightmares ☐ -anxiety ☐ -fear of surgery ☐ -sleep difficulties ☐
Were there any behavioral changes 1 month after surgery?	-Irritability ☐ -repetitive nightmares ☐ -anxiety ☐ -fear of surgery ☐ -sleep difficulties ☐ -Memory changes ☐

## References

[1] Hitti M (2007). Awake during surgery: how rare? WebMD. Awake During Surgery: How Rare? Published online.

[2] Shosholcheva M, Jankulovski N, Kuzmanovska B, Kartalov A (2016). Incidence of anesthetic awareness may be higher in low flow anesthesia. J Anesth Crit Care.

[3] Chung HS (2014). Awareness and recall during general anesthesia. Korean J Anesthesiol.

[4] Brice DD, Hetherington RR, Utting JE (1970). A simple study of awareness and dreaming during anaesthesia. Br J Anaesth.

[5] Mashour GA, Wang LYJ, Turner CR, Vandervest JC, Shanks A, Tremper KK (2009). A retrospective study of intraoperative awareness with methodological implications. Anesth Analg.

[6] Sandin RH, Enlund G, Samuelsson P, Lennmarken C (2000). Awareness during anesthesia: a prospective case study. Lancet.

[7] Pollard RJ, Coyle JP, Gilbert RL, Beck JE (2007). Intraoperative awareness in a regional medical system. Anesthesiology.

[8] Braithwaite HE, Payne T, Duce N (2023). Impact of female sex on anesthetic awareness, depth, and emergence: a systematic review and meta-analysis. Br J Anaesth.

[9] Sandhu K, Dash H (2009). Awareness during anesthesia. Indian J Anaesth.

[10] Pandit JJ, Andrade J, Bogod DG (2014). The 5th National Audit Project (NAP5) on accidental awareness during general anesthesia: summary of main findings and risk factors. Anesthesia.

[11] Mȩdrzycka-Dabrowska W, Dabrowski S, Gutysz-Wojnicka A, Ozga D, Wojtaszek M (2017). Unintended return of consciousness in a patient during surgery and general anesthesia. Eur Neurol.

[12] Wennervirta J, Ranta SOV, Hynynen M (2002). Awareness and recall in outpatient anesthesia. Anesth Analg.

[13] Ghoneim MM, Weiskopf RB (2000). Awareness anesthesia. Anesthesiology.

[14] Leslie K, Sleigh J, Paech MJ, Voss L, Lim CW, Sleigh C (2009). Dreaming and electroencephalographic changes during anesthesia maintained with propofol or desflurane. Anesthesiology.

[15] Leslie K, Myles PS, Forbes A, Chan MTV, Swallow SK, Short TG (2005). Dreaming during anesthesia in patients at high risk of awareness. Anesthesia.

[16] Leslie K, Skrzypek H, Paech MJ, Kurowski I, Whybrow T (2007). Dreaming during anesthesia and anesthetic depth in elective surgery patients: a prospective cohort study. Anesthesiology.

[17] Leslie K, Skrzypek H (2007). Dreaming during anesthesia in adult patients. Best Pract Res Clin Anaesthesiol.

[18] Whitlock EL, Rodebaugh TL, Hassett AL (2015). Psychological sequelae of surgery in a prospective cohort of patients from three intraoperative awareness prevention trials. Anesth Analg.

[19] Miller DR, Blew PG, Martineau RJ, Hull KA (1996). Midazolam and awareness with recall during total intravenous anaesthesia. Can J Anaesth.

[20] American Society of Anesthesiologists Task Force on Intraoperative Awareness (2006). Practice advisory for intraoperative awareness and brain function monitoring: a report by the American Society of Anesthesiologists task force on intraoperative awareness. Anesthesiology.

